# A Cyclic Peptide Epitope of an Under-Explored VEGF-B Loop 1 Demonstrated *In Vivo* Anti-Angiogenic and Anti-Tumor Activities

**DOI:** 10.3389/fphar.2021.734544

**Published:** 2021-09-29

**Authors:** Lei Wang, Meng Xu, Haofeng Hu, Lun Zhang, Fei Ye, Jia Jin, Hongming Fang, Jian Chen, Guiqian Chen, Sylvain Broussy, Michel Vidal, Zhengbing Lv, Wang-Qing Liu

**Affiliations:** ^1^ Zhejiang Provincial Key Laboratory of Silkworm Bioreactor and Biomedicine, College of Life Sciences and Medicine, Zhejiang Sci-Tech University, Hangzhou, China; ^2^ Department of Oncology, Zhejiang Xiaoshan Hospital, Hangzhou, China; ^3^ Université de Paris, CiTCoM-UMR 8038 CNRS, U 1268 INSERM, Paris, France; ^4^ Biologie du médicament, toxicologie, AP-HP, Hôpital Cochin, Paris, France

**Keywords:** VEGF, VEGFR, anti-angiogenic, loop mimetics, cyclic peptides

## Abstract

Pathological angiogenesis is mainly initiated by the binding of abnormal expressed vascular endothelial growth factors (VEGFs) to their receptors (VEGFRs). Blocking the VEGF/VEGFR interaction is a clinically proven treatment in cancer. Our previous work by epitope scan had identified cyclic peptides, mimicking the loop 1 of VEGF-A, VEGF-B and placental growth factor (PlGF), inhibited effectively the VEGF/VEGFR interaction in ELISA. We described here the docking study of these peptides on VEGFR1 to identify their binding sites. The cellular anti-angiogenic activities were examined by inhibition of VEGF-A induced cell proliferation, migration and tube formation in human umbilical vein endothelial cells (HUVECs). The ability of these peptides to inhibit MAPK/ERK1/2 signaling pathway was examined as well. On chick embryo chorioallantoic membrane (CAM) model, a cyclic peptide named B-cL1 with most potent *in vitro* activity showed important *in vivo* anti-angiogenic effect. Finally, B-cL1 inhibited VEGF induced human gastric cancer SGC-7901 cells proliferation. It showed anti-tumoral effect on SGC-7901 xenografted BALB/c nude mouse model. The cyclic peptides B-cL1 constitutes an anti-angiogenic peptide drug lead for the design of new and more potent VEGFR antagonists in the treatment of angiogenesis related diseases.

## Introduction

Angiogenesis (formation of new blood vessels) plays a crucial role during the development of certain diseases, such as cancer and age-related macular degeneration (Carmeliet, 2003; Chung and Ferrara, 2011). For example, angiogenesis provides nutrients and oxygen supply for tumoral cells proliferation as well as for tumoral cells metastasis (Hanahan and Folkman, 1996). Anti-angiogenic therapy consists in inhibition of angiogenesis to cut down the diffusion of nutrients in pathological tissues, which is a clinically proven target therapy to treat angiogenesis related diseases (Potente et al., 2011; Apte et al., 2019; Eelen et al., 2020). The principle of anti-angiogenic therapy is to inhibit the interaction of pro-angiogenic factors with their receptors, such as the interaction of vascular endothelial growth factors (VEGF) and their receptors (VEGFR), or to inhibit tyrosine kinase activity of VEGFRs and the downstream signal transduction pathways (Ferrara et al., 2003; Vasudev and Reynolds, 2014). Thus, anti-angiogenic drugs research has been mostly focused on the inhibition of VEGF/VEGFR interaction or the disruption of VEGFR downstream signal transduction.

Currently, three major types of anti-angiogenic drugs: antibodies, nucleotide aptamers and tyrosine kinase inhibitors, have been extensively used in clinical practice and achieved great benefits in the treatment of age-related macular degeneration (Ng et al., 2006) and several type of cancers, such as non-small cell lung cancer, gastric cancer and metastatic colorectal cancer (Carmeliet and Jain, 2011; Moserle et al., 2014). Tyrosine kinase inhibitors (sunitinib, sorafenib, axitinib or pazopanib) are mostly small molecules, they inhibit angiogenesis by blocking the kinase activity of intracellular domains of VEGFRs (Wilhelm et al., 2006; Faivre et al., 2007; Van Geel et al., 2012). They are oral administration drugs with good bioavailability but have low specificity and thus side effects (Aparicio-Gallego et al., 2011; Sun et al., 2013). Patients treated by tyrosine kinase inhibitors develop frequently drug resistance (Morais, 2014; Cabral et al., 2020). Antibodies, act as antagonists of VEGF or VEGFR, indirectly inhibiting intracellular protein kinase activity and downstream cell signaling. They exert anti-angiogenesis activities by blocking the interaction between VEGF and VEGFR. For example, bevacizumab targets the VEGF and ramucirumab targets the VEGFR2, both blockade VEGF-VEGFR interaction driven angiogenesis (Kong et al., 2017). Antibodies have high specificity, but also high production cost and high pharmacokinetic variability (Paci et al., 2020). Meanwhile, recent research showed that peptides, especially cyclic peptides, which have the similar action mode as antibodies, showed high target specificity as well as good bioavailability and metabolic stability (Fosgerau and Hoffmann, 2015; Henninot et al., 2018; Choi and Joo, 2020). Thus, development of synthetic peptide as antagonists of the VEGF/VEGFR interaction become an attractive research topic.

**FIGURE 1:**
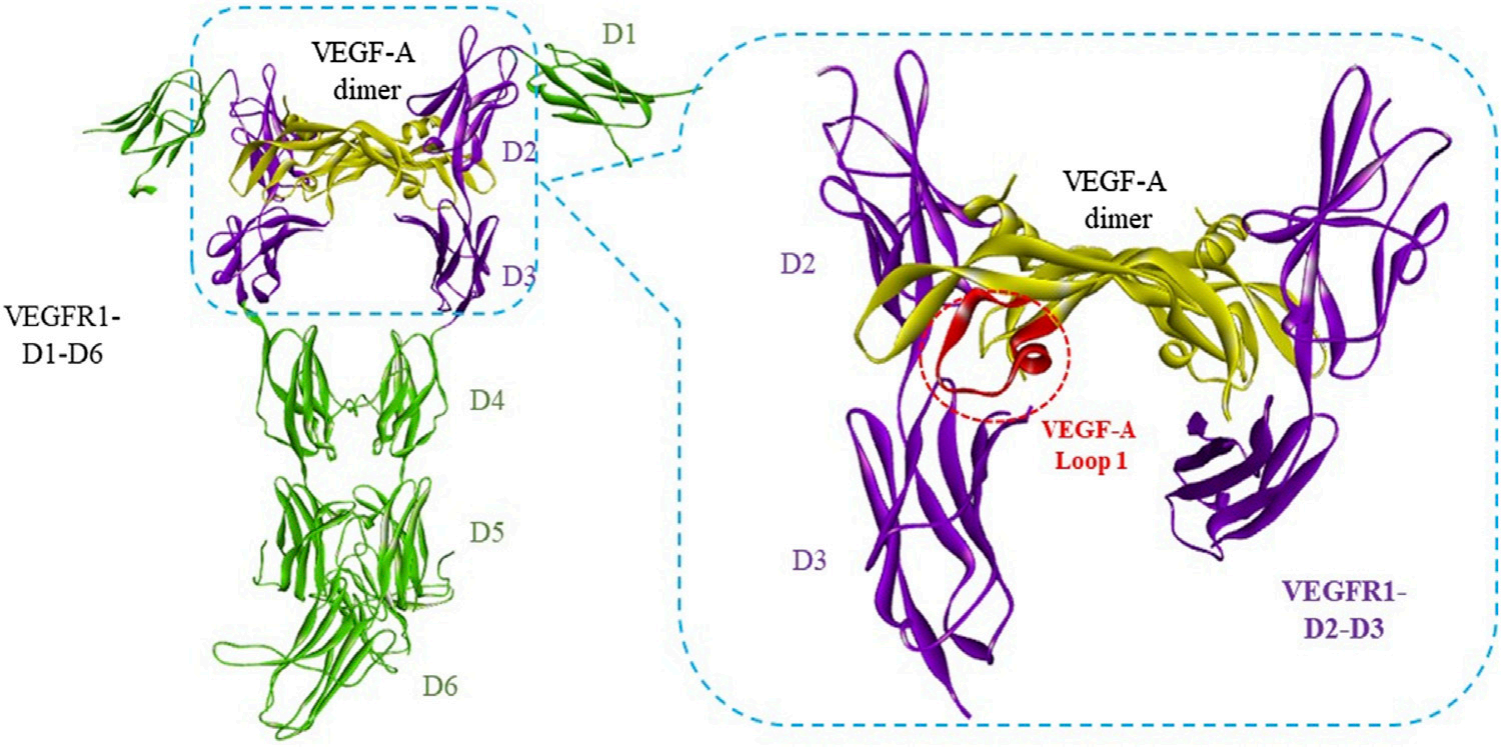
Interaction between VEGF-A with VEGFR1 D1 to D6. The complex structure of VEGF-A dimer with VEGFR1-D1-D6 was adopted from PDB 5T89 (Markovic-Mueller et al., 2017). The VEGF-A/VEGFR binding regions were framed in blue dash lines. Loop 1 of VEGF-A, circled in red dash lines, binds mainly to VEGFR1-D3.

Two major strategies are involved to develop peptide antagonists of the VEGF/VEGFR interaction: rational design or random screen of peptide libraries. Rational design of peptide antagonists is based on the complex structures of VEGF/VEGFR. VEGFs, especially VEGF-A, was reported as a key pro-angiogenic factor, which binds to membrane receptors including VEGFR1, VEGFR2, and NRP1 to exert the angiogenesis activity (Ferrara et al., 2003; Shibuya and Claesson-Welsh, 2006; Shibuya, 2014). VEGFR1 and VEGFR2 consist of seven extracellular immunoglobulin (Ig)-like domains (D1−D7), the deletion analysis showed that VEGF-A binds both on the second (D2) and the third domains (D3) of VEGFR1 (Wiesmann et al., 1997). The first structurally determined interaction between VEGF-A and VEGFRs was a complex between VEGF-A dimer and unique D2 of VEGFR1 (PDB: 1FLT) (Wiesmann et al., 1997). Numerous peptide inhibitors of VEGFRs have been designed based on this complex (Goncalves et al., 2007a; Basile et al., 2011; Garcia-Aranda et al., 2013; Wang et al., 2014). Later, more complete structures of interactions between VEGF-A and VEGFRs have been resolved, for example, a complex of VEGF-A with VEGFR2 D2-D3 (PDB: 3V2A) (Brozzo et al., 2012) and a complex of VEGF-A with VEGFR1 D1-D6 (PDB: 5T89) (Markovic-Mueller et al., 2017). These complexes showed that VEGF-A binds also partly to the third domain (D3) of VEGFR1 and VEGFR2. For example, the loop 1 of VEGF-A mainly binds to VEGFR1 D3 (Figure 1). Meanwhile, many other interaction structures of different growth factors with VEGFRs have been reported, but only with D2 of VEGFR1, such as placental growth factor (PlGF) dimer with D2 of VEGFR1 (PDB code 1RV6) (Christinger et al., 2004) and VEGF-B dimer with D2 of VEGFR1 (PDB: 2XAC) (Iyer et al., 2010). All these structure data promoted the rational design of peptide inhibitors targeting VEGF or VEGFRs for the purpose of blocking VEGF/VEGFR interaction to inhibit angiogenesis (Basile et al., 2011; Garcia-Aranda et al., 2013; Assareh et al., 2019), including our works (Goncalves et al., 2007a; Gautier et al., 2010; Wang et al., 2014; Reille-Seroussi et al., 2015; Wang et al., 2017; Wang et al., 2019).

We have recently reported the design of three cyclic peptides to mimic Loop 1 of VEGF-A, VEGF-B, and PlGF, which bind mainly to D3 of VEGFR1 and VEGFR2 according to the crystal structure data (Wang et al., 2017). These cyclic peptides showed competitive binding with VEGF to VEGFR1 and a primary tube formation inhibition in human umbilical vein endothelial cells (HUVECs) (Wang et al., 2017). In this study, we performed docking and binding energy calculation of these three cyclic peptides (named A-cL1, B-cL1, and P-cL1) with VEGFR1 to investigate the structure-activity relationship. We also examined the *in vitro* activities by evaluation of their inhibitory activity in the VEGF-A induced HUVECs proliferation, migration and tube formation by inhibiting MAPK/ERK1/2 signaling pathway. Peptide B-cL1, which showed the most important *in vitro* anti-angiogenic activity, inhibits human gastric cancer SGC-7901 cells proliferation. At last, B-cL1 was evaluated on chick embryo chorioallantoic membrane (CAM) model and human gastric cancer SGC-7901 cells xenografted BALB/c nude mouse model for its *in vivo* anti-angiogenic and antitumoral activity.

## Results

### Docking With VEGFR1

VEGF-A, VEGF-B, and PlGF are high sequence homology ligands of VEGFR1, they bind similarly to VEGFR1 according to the structure data (PDB: 1FLT, 2XAC, and 1RV6). It has been then demonstrated that Loop 1 of VEGF-A binds mainly to D3 of VEGFR1 (Markovic-Mueller et al., 2017) and VEGFR2 (Brozzo et al., 2012). In our previous study, we designed peptides mimicking different binding epitopes of VEGF-A, VEGF-B, and PlGF (Wang et al., 2017). Cyclic peptides derived from Loop 1 showed very interesting inhibition activity to disrupt the interaction of VEGF-A/VEGFR1 in a VEGF-A/VEGFR1 interaction-based ELISA assay (Table 1). Although most of reported inhibitors target VEGFR1 D2, Wiesmann et al had shown by domain deletion analysis that the deletion of D3 from VEGFR1 D1-D2-D3 induce a loss of more than 20-fold VEGF-A binding affinity (Wiesmann et al., 1997). In our reported study, cyclic peptides derived from Loop1: A-cL1, B-cL1, and P-cL1 showed higher activity than those derived from the helix α1 or the loop 2 or the loop 3. We thus focus this study on these three peptides (sequences in Table 2, formulas in Supplementary Scheme S1).

**TABLE 1:** Peptide ID, original Loop 1 sequence and derived cyclic peptide sequence. IC_50_ were determined in an ELISA based on the VEGF-A/VEGFR1 interaction.

Peptide ID	Original loop 1 sequence	Cyclic peptide sequence	IC_50_ (μM) determined by ELISA [37]
A-cL1	(VEGF-A) _36_ FQEYPDEIEYIFK_48_	[CQEYPDEIEYIC]K	50.4 ± 11.5
B-cL1	(VEGF-B) _35_ LTVELMGTVAKQLVPS_50_	Ac-[CTVELMGTVAKQLVPC]	10.4 ± 2.8
P-cL1	(PlGF) _44_ VSEYPSEVEHMFS_56_	[CSEYPSEVEHMC]S	56.0 ± 11.4

**TABLE 2:** The interaction analysis of peptide A-cL1, B-cL1, and P-cL1 with VEGFR1 according to the docking.

Type of interaction	A-cL1 [_1_CQEYPDEIEYIC]K_13_	B-cL1 Ac-[_1_CTVELMGTVAKQLVPC_16_]	P-cL1 [_1_CSEYPSEVEHMC]S_13_
Hydrogen bonds	P_5_ (O) with ARG_280_ (HN); E_9_ (O) with GLN_263_ (HNE2)	None	P_5_ (O) with ARG_280_ (HN); E_9_ (O) with GLN_263_ (HNE2)
Hydrophobic effects	I_8_ with VAL_278_, PHE_292_; I_11_ with HIS_223_, ARG_261_; K_13_ with ILE_145_, HIS_223_	V_9_ with ILE_145_; A_10_ with HIS_223_; K_11_ with ARG_261_	V_8_ with VAL_278_, ARG_280_, PHE_292_

**FIGURE 2:**
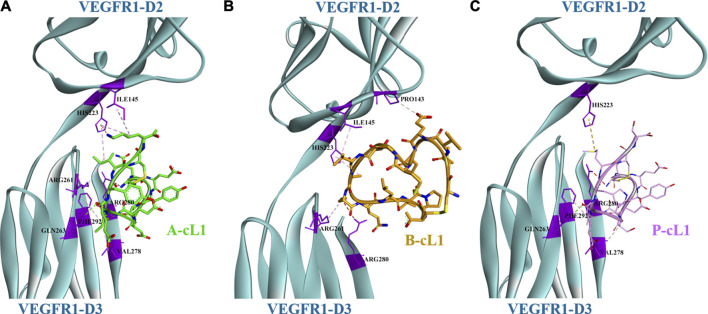
Docking model of peptides A-cL1, B-cL1, and P-cL1 with VEGFR1 D2-D3. Binding residues on VEGFR1 D2-D3 are in purple; hydrogen bonds labelled in green dash lines; hydrophobic effects labelled in violet dash lines. **(A)** Binding between A-cL1 (green) and VEGFR1 D2-D3 (turquoise), **(B)** Binding between B-cL1 (orange) and VEGFR1 D2-D3 (turquoise); **(C)** Binding between P-cL1 (pink) and VEGFR1 D2-D3 (turquoise).

Peptides A-cL1, B-cL1, and P-cL1 were respectively designed from Loop 1 of VEGF-A, VEGF-B, and PlGF, which were supposed to bind mainly to the domain 3 (D3) of VEGFR1. The docking results showed that A-cL1 and P-cL1 had similar interaction mode with VEGFR1, mainly binding to VEGFR1 D3 (Figures 2A,C). However, B-cL1, which had larger cycle, binds differently to VEGFR1. B-cL1 binds to the interface of VEGFR1 D2 and D3 (Figure 2B). The ten highest score peptide structures were superimposed in the binding with VEGFR1 (Supplementary Figures S1–S3). The major interactions between A-cL1, B-cL1, P-cL1, and VEGFR1 were analyzed according to the docking (Table 2).

### Inhibition of HUVECs Proliferation, Migration and Tube Formation

VEGFR1 and VEGFR2 have very similar structures. The crystal structure of VEGF-A/VEGFR1 D1-D6 (PDB: 5T89) and the crystal structure of VEGF-A/VEGFR2 D2-D3 (3V2A) showed the same binding epitopes on VEGF-A, which suggests that the epitope mimic peptides of VEGF-A are normally able to bind both VEGFR1 and VEGFR2. An *in vitro* inhibitor selection assay was previously developed in our laboratory based on the interaction of VEGF-A/VEGFR1 (Goncalves et al., 2007b). Inhibitors selected from this assay that are able to inhibit the interaction of VEGF-A/VEGFR1, should probably inhibit as well as the interaction of VEGF-A/VEGFR2 and their downstream signaling. The investigation of A-cL1, B-cL1, and P-cL1 induced *in vitro* anti-angiogenic effects was performed by cell proliferation, cell migration and tube formation in HUVECs.

#### Inhibition of HUVECs Proliferation

**FIGURE 3:**
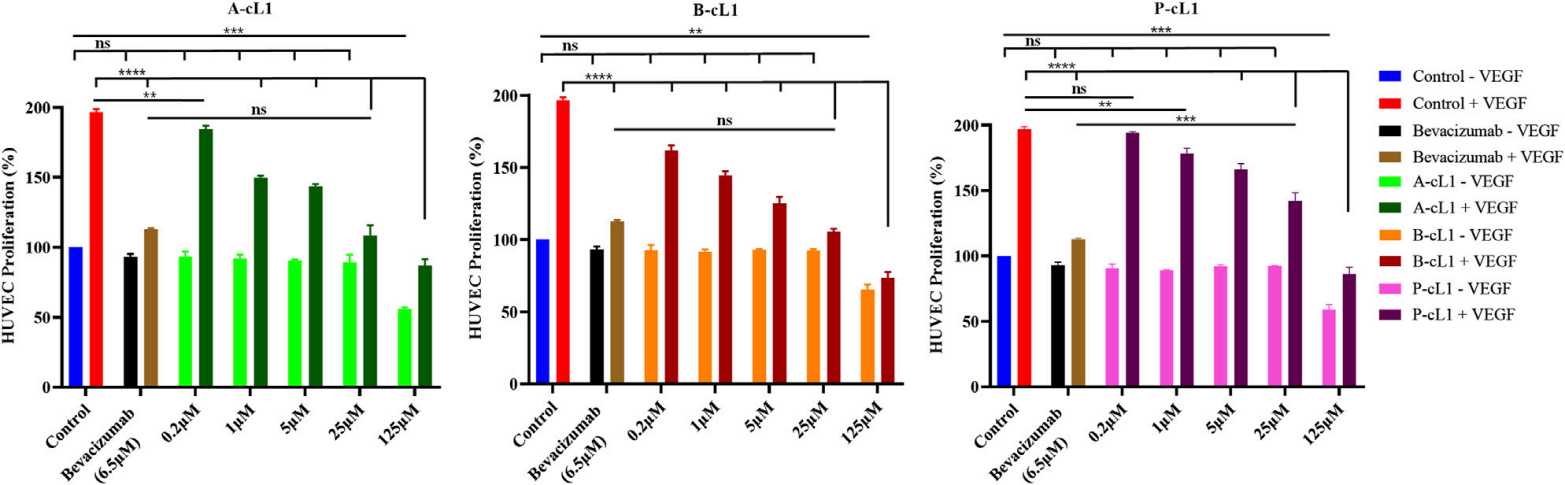
Peptides A-cL1, B-cL1, and P-cL1 inhibit VEGF-A stimulated HUVECs proliferation. HUVECs were cultured in serum-free medium without or with 0.2 μg/ml VEGF-A (negative control -VEGF and positive control + VEGF), treated with 6.5 μM bevacizumab without or with VEGF; treated with 0.2, 1, 5, 25, and 125 μM of A-cL1, B-cL1 or P-cL-1 without or with VEGF. The relative cell proliferation (%) were analyzed with GraphPad Prism 8 (mean ± SEM of three independent experiments), compared to the control group in one-way ANOVA statistical analysis. *p < 0.05, **p < 0.01, ***p < 0.001, ****p < 0.0001, “ns” meaning non-significant.

As shown in Figure 3, additional VEGF-A (0.2 μg/ml) in culture medium stimulated HUVECs proliferation (control group). Bevacizumab (Avastin®), a monoclonal antibody drug targeting VEGF-A to block the interaction of VEGF-A with VEGFRs, was tested as positive control. At 6.5 μM (1 mg/ml), bevacizumab resulted in strong inhibition of VEGF-A induced HUVECs proliferation. Peptide A-cL1, B-cL1, and P-cL1 showed a dose-dependent inhibition of HUVECs proliferation at five concentrations (0.2, 1, 5, 25, and 125 μM) (Figure 3). A-cL1 and B-cL1 showed better inhibition of HUVECs proliferation than P-cL1. They showed similar inhibition activity at 25 μM as bevacizumab at 6.5 μM, while B-cL1 exhibited more effective inhibition at 0.2 μM than A-cL1. All the three peptides showed slight cytotoxicity at 125 μM.

#### Inhibition of HUVECs Migration

**FIGURE 4:**
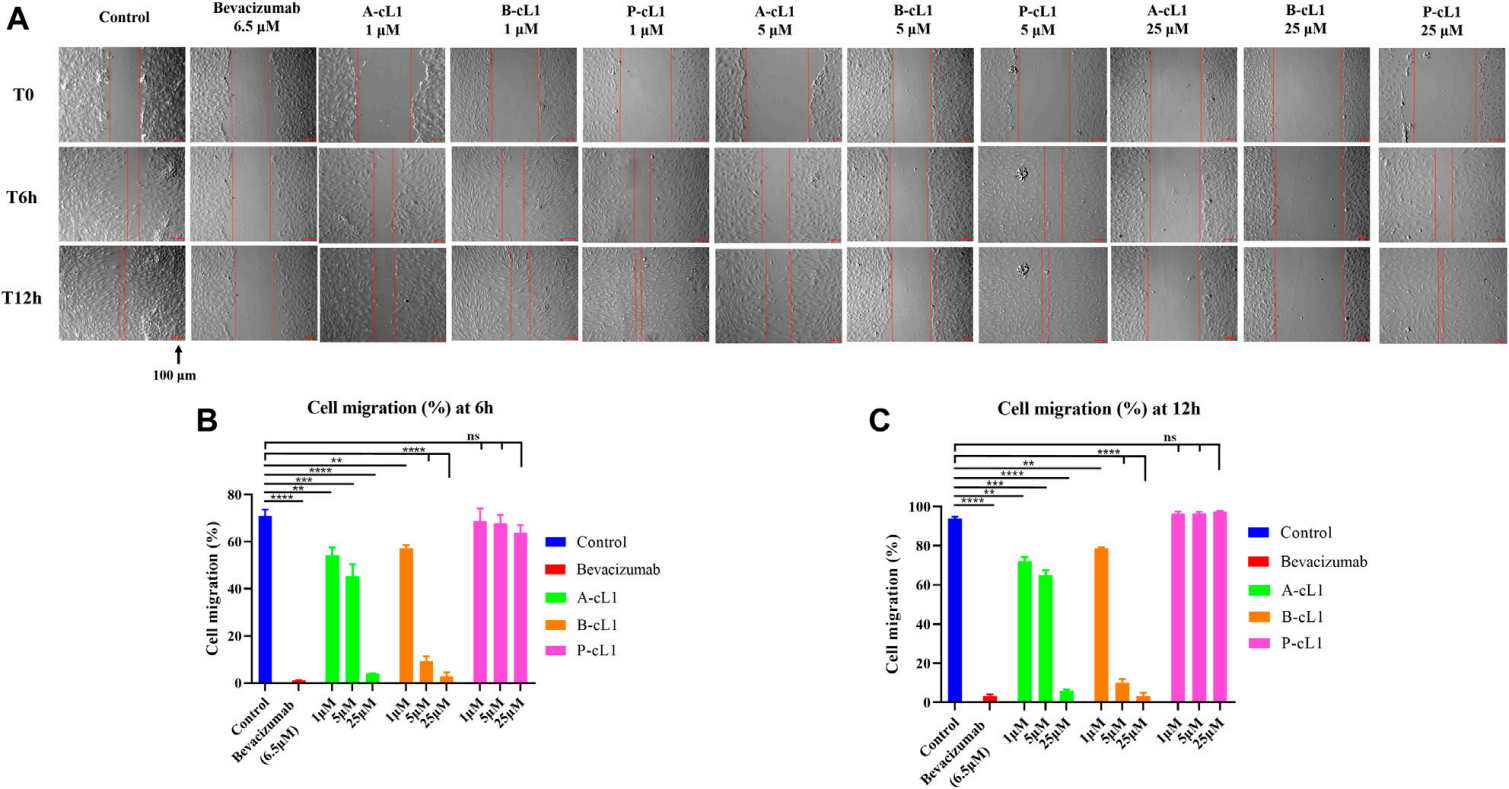
A-cL1, B-cL1, and P-cL1 inhibit VEGF-A (0.2 μg/ml) stimulated HUVECs migration. **(A)** Representative images of scratch wound-healing assay. After creation of scratches, HUVECs were cultured in 2% serum medium containing 0.2 μg/ml VEGF-A, treated with serum-free medium (control group, blue), or with 6.5 µM bevacizumab (positive control, red), or with 1 μM, 5 and 25 μM of A-cL1, B-cL1 or P-cL1. Images were taken before the treatment (T0h), after 6 h treatment (T6h) and 12 h treatment (T12h). Scale bar, 100 μm. **(B)** Quantitative analysis of the cell migration (%) by measuring wound closure area after 6 h treatment using ImageJ. **(C)** Quantitative analysis of the cell migration (%) by measuring wound closure area after 12 h treatment using ImageJ. The relative cell migration (%) were analyzed with GraphPad Prism 8 (mean ± SEM of three independent experiments), compared to the control group in one-way ANOVA statistical analysis. *p < 0.05, **p < 0.01, ***p < 0.001, ****p < 0.0001, “ns” meaning non-significant.

The inhibition of VEGF-A stimulated cell migration by A-cL1, B-cL1, and P-cL1 was evaluated by a wound healing assay (Figure 4). After stimulation with VEGF-A (0.2 μg/ml), HUVECs were able to migrate through the scratched area, and completely fill the scratched area after 12 h (Figure 4, control group). Bevacizumab (6.5 μM) resulted in efficient inhibition of HUVECs migration. After treatment with A-cL1, B-cL1, and P-cL1, HUVECs migration was inhibited. B-cL1 and A-cL1 induced dose-dependent inhibition of HUVECs migration. B-cL1 was able to inhibit strongly HUVECs migration at 5 μM, less than 10% of migration were observed at both 6 and 12 h. A-cL1 inhibited partially HUVECs migration at 5 μM, but it could also completely inhibit HUVECs migration at 25 μM. P-cL1 showed a week inhibition of HUVECs migration at 6 h even at 25 μM, HUVECs filled up the scratched area after 12 h treatment of P-cL1 at the three concentrations.

#### Inhibition of HUVECs Tube Formation

**FIGURE 5:**
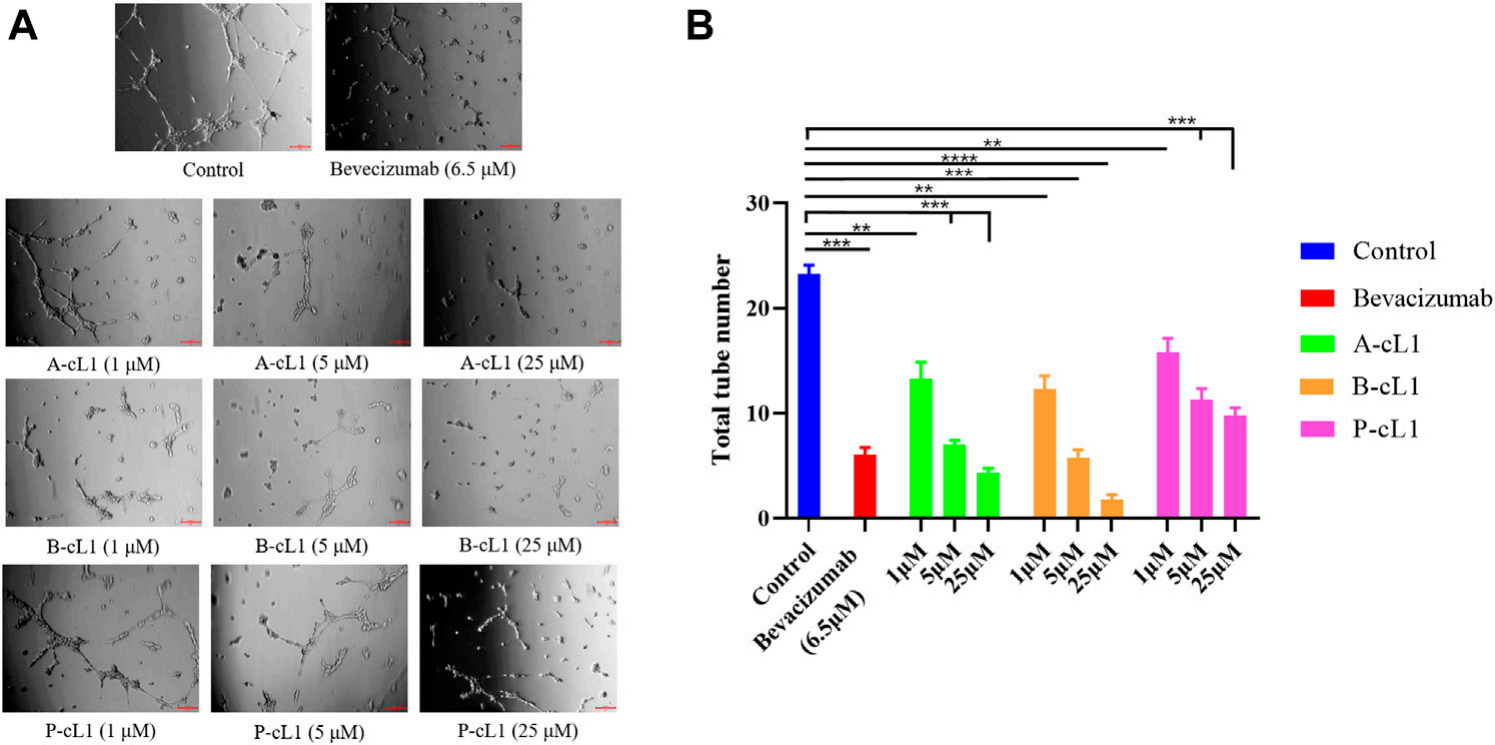
A-cL1, B-cL1, and P-cL1 inhibit VEGF-A stimulated tube formation in HUVECs. **(A)** Representative images of the capillary tube formation assay. After seeded on Matrigel® HUVECs were cultured in 2% serum medium containing 0.2 μg/ml VEGF-A, treated with serum-free medium (control group), or with bevacizumab (positive control), or with 1 μM, 5 and 25 μM of A-cL1, B-cL1 or P-cL1. Images were taken after 6 h treatment. **(B)** Quantitative analysis of the tube formation by counting the capillary number. Data are presented as mean ± SEM of three experiments, statistics analysis using GraphPad Prism 8, compared to the control group in one-way ANOVA. ***p < 0.001, ****p < 0.0001.

Capillary-like tube formation is one of the most important pseudo angiogenic test using HUVECs. In this test, two-dimensional tube formation was stimulated by additional VEGF-A (0.2 μg/ml) on Matrigel®. A-cL1, B-cL1, and P-cL1 were tested at three concentrations (1 μM, 5 and 25 μM) for their ability to inhibit VEGF-A stimulated tube formation in HUVECs. The total numbers of formed tubes were measured (Figure 5). All the three peptides showed a dose-dependent inhibition of tube formation in HUVECs. B-cL1 was able to inhibit significantly HUVECs tube formation at 25 μM, and it had an equal inhibition activity at 5 μM as bevacizumab (6.5 μM). A-cL1 also exhibited a good inhibition of VEGF-A stimulated tube formation, it had a similar inhibition activity at 25 μM as bevacizumab (6.5 μM). P-cL1 inhibited VEGF-A stimulated tube formation in HUVECs in a dose-dependent manner with a weaker activity than A-cL1 and B-cL1, short capillary-like structures were still observed after treatment with P-cL1 at 25 μM.

These results showed that peptides A-cL1, B-cL1, and P-cL1, derived from epitope Loop 1 of VEGF-A, -B, and PlGF can inhibit VEGF-A/VEGFRs interaction induced migration and tube formation in HUVECs. B-cL1 showed the highest inhibitory activity, while P-cL1 showed the lowest activity among the three peptides. The results are in agreement with peptides IC_50_ determined in a VEGF/VEGFR1 interaction-based ELISA assay (Wang et al., 2017) (Table 1).

### Inhibition of Intracellular Signaling Pathways Associated With VEGF

**FIGURE 6:**
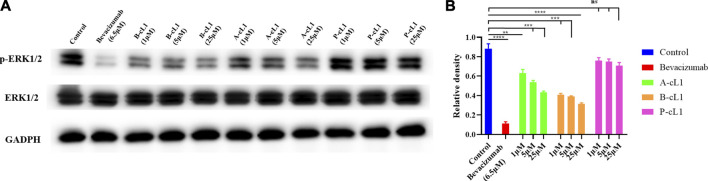
A-cL1, B-cL1, and P-cL1 inhibit p-ERK1/2 formation by inhibiting VEGF/VEGFRs interaction. **(A)** Western blots using p-ERK1/2 and total ERK1/2 extracts of HUVECs after treatment either with A-cL1, B-cL1 or P-cL1 (1 μM, 5 and 25 μM), or with bevacizumab (6.5 μM) as positive control, or with serum-free medium (control group). GAPDH was used as loading control. **(B)** Quantification of relative density of p-ERK1/2 and total ERK1/2 were analyzed by ImageJ. The bar chart illustrates relative density values that analyzed using GraphPad Prism 8. Data are presented as mean ± SEM of three experiments, compared to the control group in one-way ANOVA statistical analysis. *p < 0.05, **p < 0.01, ***p < 0.001, ****p < 0.0001, “ns” meaning non-significant.

A-cL1, B-cL1, and P-cL1 having different VEGFR1 binding affinity showed dose-dependent different inhibitory effects in HUVECs migration and tube formation. In order to verify whether the different activity of A-cL1, B-cL1, and P-cL1 could result in different intensity of downstream signal transduction, we performed western blot analysis of the p-ERK1/2 level in the MAPK/ERK1/2 signaling pathway, which is a proven signal pathway induced by the interaction of VEGF/VEGFRs (Shibuya, 2011). After HUVECs incubation with A-cL1, B-cL1, and P-cL1 at different concentrations (1 μM, 5 and 25 μM) in the presence of VEGF-A (0.2 μg/ml), the p-ERK1/2 level was determined in cell lysate. As shown in Figure 6, bevacizumab (6.5 μM) had the most important inhibition of p-ERK1/2 formation. A-cL1 and B-cL1 significantly decreased the level of p-ERK1/2 in a dose-dependent manner, while P-cL1 showed a limited decrease of p-ERK1/2 formation compared to the control.

### B-cL1 Inhibits Angiogenesis on Chick Embryo Chorioallantoic Membrane Model

**FIGURE 7:**
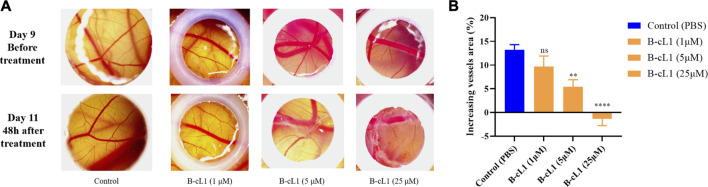
B-cL1 inhibits angiogenesis in CAM model. **(A)** Representative images of chorioallantoic membrane before and after treatment (48 h) with B-cL1 at 1 μM, 5 and 25 μM, or with PBS as control. **(B)** The percentage of capillary area in total analyzed area was quantified with AngioTool (Zudaire et al., 2011), the difference of vessels area (percentage of capillary area at T48h - percentage of capillary area at T0h) was analyzed using GraphPad Prism 8. Data are presented as mean ± SEM of three experiments, compared to the control group in one-way ANOVA statistical analysis. **p < 0.01, ****p < 0.0001, “ns” meaning non-significant.

Among the three peptides, B-cL1 showed the most important *in vitro* anti-angiogenic activity. In order to further study the *in vivo* anti-angiogenic activity of B-cL1, we performed an evaluation in a chick embryo chorioallantoic membrane (CAM) model, which is a common physiological and pathological angiogenesis study model *in vivo* (Rezzola et al., 2020). As shown in Figure 7A, on day 9, capillaries are well formed on the surface of the chick embryo chorionic epithelium. After treatment with different concentrations of B-cL1 (1 μM, 5 and 25 μM) for 48 h, formation of new capillaries was inhibited compared with control treated by PBS (Figure 7A). At 1 μM of B-cL1, short capillaries formation was still observed. However, the increase in thickness of blood vessels was inhibited. At 5 μM of B-cL1, both the formation of new capillaries and the development of blood vessels thickness were inhibited. At 25 μM of B-cL1, destruction of pre-existing capillaries was observed. AngioTool (Zudaire et al., 2011), a software for quantitative analysis of angiogenesis, which allows to quantifier the percentage of capillary area in total area (Klotz et al., 2015; Sawaguchi et al., 2017), was used to analyze the increasing or decreasing vessels area after treatment. The results showed that B-cL1 was able to inhibit new capillaries formation in a dose-dependent manner (Figure 7B).

### B-cL1 Inhibits Human Gastric Cancer SGC-7901 Cell Proliferation and Tumor Growth on Human Gastric Cancer Xenografted BALB/c Nude Mouse Model

Gastric cancer is one of the most common malignancy that threaten the health of human beings, and the human gastric cancer SGC-7901 cell line is widely used in anti-tumoral studies in cellular level and on xenografted mouse model (Lv et al., 2019). This kind of tumor is largely explored in clinical trial by anti-angiogenic approach, including anti-VEGF, which have shown some benefits in second line treatment (Park et al., 2015). Consequently, anti-tumoral activity of B-cL1 was evaluated by VEGF-induced (50 ng/ml) human gastric cancer SGC-7901 cell proliferation and on BALB/c mice subcutaneous xenograft of human gastric cancer SGC-7901 cells.

**FIGURE 8:**
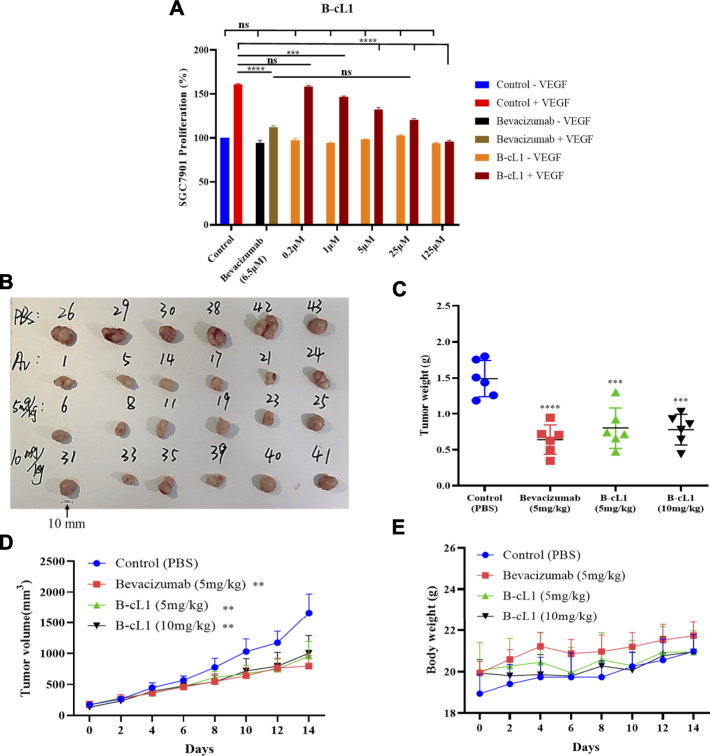
B-cL1 inhibits human gastric cancer SGC-7901 cell proliferation and tumor growth on BALB/c nude mice orthotopic transplantation model. **(A)** Human gastric cancer SGC-7901 cells were cultured in serum-free medium without or with 50 ng/ml VEGF (negative control − VEGF; positive control + VEGF), treated with 0.2, 1, 5, 25 and 125 μM of B-cL1 without or with 50 ng/ml VEGF; or treated with 6.5 μM bevacizumab without or with 50 ng/ml VEGF. The relative cell proliferation (%) were analyzed with GraphPad Prism 8. **(B)** Images of tumors separated from mice (numbers represent randomized mouse number) after 14 days’ treatment with B-cL1 (5 mg/kg/day and 10 mg/kg/day), or bevacizumab (Av, 5 mg/kg, once), or PBS as control. **(C)** Quantification of tumor weight. **(D)** Quantification of tumor volume every 2 days. **(E)** Quantification of mice body weight every 2 days. Data are presented as mean ± SD, compared to the control group in one-way ANOVA statistical analysis. *p < 0.05, **p < 0.01, ***p < 0.001, ****p < 0.0001.

B-cL1 showed a dose-dependent inhibition of human gastric cancer SGC-7901 cell proliferation at five concentrations (0.2, 1, 5, 25 and 125 μM), no cytotoxicity was observed event at 125 μM (Figure 8A). It showed similar anti-proliferation activity at 25 μM as bevacizumab at 6.5 μM. Meanwhile, B-cL1 (5 mg/kg/day and 10 mg/kg/day) and bevacizumab (5 mg/kg, only once) were intravenously administrated for 2 weeks on BALB/c nude mice subcutaneous xenograft of SGC-7901 cells, starting when the tumor volume arrived at 150–300 mm^3^. Tumor volume and mice body weight were measured every 2 days. After 14 days of treatment, the mice were euthanized using CO_2_ followed by cervical dislocation to ensure death and the tumors were separated (Figure 8B). The results showed that B-cL1 (5 mg/kg/day) reduced 60% of tumor weight, 53% of tumor volume, compared to control group; B-cL1 (10 mg/kg/day) showed 62% reduction of tumor weight, 51% reduction of tumor volume, compared to control group. As a positive control, bevacizumab (5 mg/kg) reduced 57% of tumor weight and 57% of tumor volume with only one administration (Figures 8C,D). It is worth to indicate that no mortality of mice was observed during the 2 weeks of treatment, and the body weights of mice was increased reasonably (Figure 8E), which suggest weak toxicity of B-cL1 for an administration up to 10 mg/kg/day.

## Discussion

Blocking VEGF/VEGFRs interaction is a strategy to treat angiogenesis related diseases. Antibody drugs, such as bevacizumab, ramucirumab, ranibizumab, and fusion protein, such as aflibercept, can bind either VEGF or VEGFRs to inhibit VEGF/VEGFRs interaction (Lazzeri et al., 2015; Eelen et al., 2020). They have been approved in clinical treatment of multitype of cancers or neovascular (wet) age-related macular degeneration (AMD). Current research of peptide-based inhibitors for VEGF/VEGFRs interaction mainly start with rational design or random screen of peptide libraries targeting mainly the D2 of VEGFRs or the VEGF (Basile et al., 2011; Garcia-Aranda et al., 2013; De Rosa et al., 2014; Bayo-Puxan et al., 2016). They have shown ability to inhibit VEGF/VEGFRs interaction, and some of them showed *in vitro* activities. However, their *in vivo* activities were less reported. Previously, we have designed several series of peptides mimicking different VEGFRs binding epitopes (helix and loops) of VEGF-A, VEGF-B, and PlGF (Goncalves et al., 2007a; Gautier et al., 2010; Wang et al., 2014; Wang et al., 2017; Wang et al., 2019). Three peptides (A-cL1, B-cL1, and P-cL1) respectively mimicking Loop 1 of VEGF-A, VEGF-B or PlGF showed the most important inhibition of VEGF-A/VEGFRs interaction in an *in vitro* competition assay (Wang et al., 2017). Here, we performed VEGF-A induced cell migration and tube formation assays in HUVECs, followed by western blot analysis of VEGFRs downstream signaling studying ERK activation. We also performed the evaluation of physiological anti-angiogenesis activity in chick embryo chorioallantoic membrane (CAM) model. Finally, we evaluated the inhibition of human gastric cancer SGC-7901 cells proliferation and tumor growth activity in SGC-7901 cells subcutaneous xenograft model on BALB/c nude mice. All these experiments allowed us to investigate the *in vitro* and *in vivo* anti-angiogenesis activity as well as anti-tumor activity of this series of cyclic peptides derived from under-explored Loop 1.

The docking results identified the different binding sites on VEGFR1 of A-cL1, B-cL1, and P-cL1. A-cL1, and P-cL1 bind similarly as the original Loop 1 structure of VEGF-A and PlGF to the domain 3 (D3) of VEGFR1. However, B-cL1 with a larger cycle binds differently. It binds to the interface of VEGFR1 D2-D3 with higher flexibility (Supplementary Figure S2). VEGF-A was reported to bind tightly to VEGFR1 D1-D7. The deletion of D1 or D4-D7 had slight influence on VEGF binding affinity, the deletion of D3 could cause a 25-fold diminution of binding affinity. However, D2 stays as the major binding domain of VEGF-A (Wiesmann et al., 1997). A-cL1 and P-cL1 binding majorly to D3 had higher IC_50_ (50 and 56 µM respectively), whereas B-cL1 binding to both D2 and D3 had lower IC_50_ (10 µM) (Table 1) (Wang et al., 2017). The docking results showed that targeting only D3 is insufficient to inhibit VEGF/VEGFR interaction.

The *in vitro* anti-angiogenic activities of A-cL1, B-cL1, and P-cL1 were evaluated in VEGF-induced HUVECs proliferation, migration and tube formation. The results showed that their different inhibitory activity was correlated to their IC_50_ values of VEGF-A/VEGFR1 interaction inhibition (Table 1). B-cL1, having the lowest IC_50_ value (10 μM) measured in a VEGF/VEGFR1 interaction-based ELISA assay, demonstrated the most potent inhibition in VEGF-induced HUVECs proliferation, migration and tube formation. Moreover, B-cL1 showed the most potent inhibitory effect on VEGF-induced VEGFRs signaling through MAPK/ERK1/2 pathways. These results showed that B-cL1, designed from Loop 1 of VEGF-B, had very important *in vitro* anti-angiogenic activity by binding to VEGFRs to inhibit VEGF-A/VEGFRs interaction, thus inhibiting VEGFRs signal transport.

The *in vivo* evaluation of B-cL1 in CAM model showed that B-cL1was able to inhibit physiological angiogenesis on the surface of chorionic epithelium during the chick embryo development at 5 μM, and more significantly at 25 μM.

The anti-tumor activities of B-cL1 were then studied. B-cL1 was able to inhibit *in vitro* in a dose-dependent way VEGF-induced human gastric cancer SGC-7901 cells proliferation. In SGC-7901 cells subcutaneous xenograft BALB/c mouse model, intravenous administration of bevacizumab (5 mg/kg, just once injection for 2 weeks) significantly inhibit tumor growth. Bevacizumab, as recommended, was injected by intravenous every 14 days at the dose of 5 mg/kg (Garcia et al., 2020). However, peptides are commonly considered with poor *in vivo* stability (Diao and Meibohm, 2013; Henninot et al., 2018). Thus, we performed *in vivo* evaluation of cyclic peptide B-cL1, at doses 5 mg/kg/day and 10 mg/kg/day. The results showed similar tumor growth inhibition as bevacizumab at 5 mg/kg. No obvious toxicity of B-cL1 was observed up to 10 mg/kg/day. Interestingly, non-significant difference in tumor growth inhibition was observed for administration of 5 mg/kg/day and 10 mg/kg/day of B-cL1, which suggests that 5 mg/kg/day of B-cL1 can induce maximal tumor growth inhibition. The tumor growth inhibition observed *in vivo* is probably correlated with the inhibition of ERK1/2 phosphorylation observed *in vitro* on HUVE cells. Which may be the result of the inhibition of VEGF-VEGFR interaction. Recently, the group of S. M. Asghari has designed a series of peptides derived from VEGF-A and VEGF-B epitopes binding to D2 of VEGFR. The peptides named VGB (Sadremomtaz et al., 2018), VGB1 (Assareh et al., 2019), VGB4 (Farzaneh Behelgardi et al., 2018), and VGB3 (Sadremomtaz et al., 2020) were tested in murine mammary carcinoma tumor model (MCT) where BALB/c mice were implanted with murine breast cancer 4T1 cells. In VEGFR1-dependent MCT model, the peptides exhibited inhibition of tumor growth at doses of 5 mg/kg, with peptide VGB3 displaying effects at a very low dose of 0.2 mg/kg. Therefore, investigation of the peptide B-cL1 on tumor models characterized by varied expression levels of VEGFR1 and VEGFR2 will be of interest to determine its anti-angiogenesis activity profile more accurately. Otherwise, our results, coupled with those of the group of S. M. Asghari, show a relative stability of cyclic peptides in physiological medium.

Peptide antagonists reported till now are all D2 targeting molecules except this series of cyclic peptides A-cL1, B-cL1, and P-cL1. In this study, we determined by molecular docking that B-cL1 binds D2 and D3 simultaneously, but A-cL1 and P-cL1 bind mainly to D3. We confirmed that B-cL1 has higher anti-angiogenic activities than A-cL1 and P-cL1. The results open a new way to develop VEGFR inhibitors. Indeed, the conception of Aflibercept, consisting of VEGFR1-D2 and VEGFR2-D3 fused to human IgG1 Fc portion (Lazzeri et al., 2015), supports the importance of targeting both D2 and D3 of VEGFR in the research of VEGFR inhibitors.

## Materials and Methods

### Peptide Inhibitors

The design and synthesis of peptides derived from different epitopes of VEGFR1 binding sites on VEGF-A, VEGF-B, and PlGF have been reported in our previous work (Wang et al., 2017). Briefly, to mimic the loop structure, the original peptide sequences have been modified and then cyclized through disulfide bonds. To facilitate the reading, we rename these peptides (peptide 14, 18, and 19 in previous work) as described in the Table 1.

### Docking With VEGFR1

Protein Preparation Wizard Workflow program (Gayatri et al., 2016) provided in Maestro 9.0 was used to build the structures of peptides A-cL1, B-cL1, and P-cL1 based on the original crystal structures of VEGF-A, VEGF-B, and PlGF with VEGFR1-D2 (PDB code: 1FLT, 2XAC, 1RV6). Peptides were built by mutating underlined residues to cysteine residues and formed disulfide bond on the side chains (Table 1). They were then aligned and docked near the binding pocket of Loop1 on VEGFR1 (using complex structure of VEGF-A with VEGFR1 D1-D6, PDB: 5T89) in Pymol to generate the initial PDB complex file of A-cL1/5T89, B-cL1/5T89 and P-cL1/5T89. Afterwards, the peptide-protein complexes were adapted to optimize the side chains, in order to eliminate internal collision that are not related to intermolecular interactions in Rosetta FlexPepDock program (Raveh et al., 2010). At last, the docking was performed with optimized structures of the three peptides on Rosetta online server (http://flexpepdock.furmanlab.cs.huji.ac.il/index.php) (Raveh et al., 2010; London et al., 2011). Ten structure models of each peptide with highest score were obtained among 200 generated structures (Supplementary Figures S1–S3) and were output for binding analysis.

### Cell Culture

Human umbilical vein endothelial cells (HUVECs), of passage 3 to 6, were purchased from Shanghai Fuheng Biotechnology Co. LTD. and cultured in endothelial cell culture medium (ECM: Gibco, Life Technologies, United States), with 10% fetal bovine serum (FBS: Gibco, Life Technologies, United States), 100 U/mL penicillin and 100 mg/ml streptomycin in an incubator at 37°C and containing 5% carbon dioxide (CO_2_). Human gastric cancer SGC-7901 cells, from Cell Bank of the Chinese Academy of Sciences (Shanghai, China), were cultured in Roswell Park Memorial Institute (RPMI) 1,640 medium (Gibco, Life Technologies, United States), with 10% fetal bovine serum (FBS: Gibco, Life Technologies, United States), 100 U/mL penicillin and 100 mg/ml streptomycin in an incubator at 37°C and containing 5% carbon dioxide (CO_2_).

### VEGF-Induced HUVECs Proliferation Assay

HUVECs (3 × 10^3^) were seeded on the 96-well plate per well in ECM containing 5% FBS, and incubated overnight at 37°C, with 5% CO_2_. Then the medium was removed. New serum-free medium containing 0.2 μg/ml VEGF-A (R&D Systems, United Kingdom) with different concentrations of peptides (0.2, 1, 5, 25 and 125 μM), bevacizumab (Roche, Switzerland) at 6.5 μM (1 mg/ml) or control group (serum-free medium) were added and incubated for another 48 h (6 wells/concentration/group). The effects of proliferation were quantified by Cell Counting Kit-8 (CCK-8, Sigma, United States) assays. The absorbance was measured at 450 nm with an enzyme-linked immunoassay reader (AMR-100, ALLSHENG, Hangzhou, China). The experiment was repeated three times.

### VEGF-Induced Wound Healing Assay in HUVECs

The six-well plate was coated with HUVECs and incubated with ECM containing 10% FBS until the confluence of monolayer cells reached 100%. A scratching wound was created with 10 μl pipette tip. Then the medium was removed. New medium containing only 2% serum and 0.2 μg/ml (5 nM) VEGF-A (R&D Systems, United Kingdom) with different concentrations of peptides (1 μM, 5 and 25 μM), bevacizumab (1 mg/ml, 6.5 μM) or 2% serum medium (control group) were added and incubated at 37°C, with 5% CO_2_ for another 12 h. Cell migration was observed and photographed at 6 and 12 h under a microscope (TE-2000U, NIKON, Japan). The results were calculated from three independent experiments with four replicates. Wound area (the open area of the scratch) was quantified using ImageJ, and the percentage of wound closure were calculated: [1 - (wound area at 6 h or 12 h/wound area at 0 h)] × 100%.

### HUVECs Tube Formation Assay

Growth factor-reduced basal membrane extract (BD Biocoat^TM^, 356,230, United States) was melted overnight in refrigerator at 4°C, and precoated on the 96-well plate. The 96-well plate was then incubated at 37°C for 1 h until Matrigel solidified. 5 × 10^4^ HUVECs/well, pre-treated with different concentrations of peptides (1 μM, 5, and 25 μM), bevacizumab (6.5 μM) or 2% serum medium (control group) were added to the plate and incubated at 37°C for 6 h. Resulting tube networks were observed and photographed by using the microscope (TE-2000U, NIKON, Japan). The numbers of cell branches were quantified in a blind manner. The results were calculated from three independent experiments with four replicates.

### Western Blot Analysis

HUVECs treated with different concentrations of peptides (1 μM, 5, and 25 μM), bevacizumab (6.5 μM) or serum-free medium (control group) were lysed with RIPA lysis buffer (Beyotime, Shanghai, China) containing protease and phosphatase inhibitors (Sigma, United States). After centrifugation at 4°C, protein concentration was determined by bicinchoninic assay (BCA) (Beyotime, Shanghai, China). Polyacrylamide gel electrophoresis was performed with the same amount of protein of different groups. The protein on the gel was then transferred to the PVDF membrane (Millipore, United States), and the membrane was blocked with skimmed milk powder in Tris-buffered saline (TBS, 5%) for 2 h. Later, the membrane was washed with TBST (1 × Tris Buffered saline, 0.1%Tween20), and incubated with the primary antibody (anti-phospho ERK1/2, anti-ERK1/2, and anti-GAPDH as control, Cell Signaling Technology, United States) and TBST overnight at 4°C. The membrane was washed 3 times with TBST, and then incubated with horseradish peroxidase-conjugated goat anti-mouse IgG secondary antibody and goat anti-rabbit IgG secondary antibody for 1 h at room temperature. At last, proteins were analyzed by ECL reagent (WesternBright ECL, United States) following the manufacturer’s protocol, after 3 times washing with TBST. The experiment was repeated three times. Protein expression was quantified by ImageJ.

### *In Vivo* Anti-Angiogenic Evaluation of B-cL1 Using the Chick Chorioallantoic Membrane Assay

A chick chorioallantoic membrane (CAM) assay was carried out to determine the *in vivo* anti-angiogenic activity of peptide B-cL1. Two-days old fertilized specific pathogen free (SPF) eggs (Ningbo Chunpai Agricultural Science and Technology Co. LTD., Ningbo, China) were incubated at 37.5°C, 60–70% relative humidity for 1 week. Blood vessels in SPF eggs were observed within lighting. Then a window of about 2 cm^2^ was opened on the eggs and a sterilized silica gel ring (1.5 cm inner diameter) was placed onto the CAM. 20 μL of different concentration of B-cL1 (1 μM, 5, and 25 μM in PBS) or PBS (control group) were added inside the silica gel ring (10 eggs per group). The window was recovered with sterilized tape, and the eggs were incubated for another 48 h. The sterilized tape was removed, and the area of capillary blood vessels photographed under a stereomicroscope (Soptop, Shanghai, China). The experiment was repeated three times, and the percentage of capillary area in total analyzed area was calculated using AngioTool (Zudaire et al., 2011). The percentage of increasing/decreasing capillary area was then calculated: percentage of capillary area at 48 h - percentage of capillary area at 0 h.

### VEGF-Induced Human Gastric Cancer SGC-7901 Cells Proliferation Assay

Human gastric cancer SGC-7901 cells (2 × 10^3^) were seeded on the 96-well plate per well in RPMI 1640 medium containing 2% FBS, and incubated overnight at 37°C, with 5% CO_2_. Then the medium was removed. New serum-free medium containing 50 ng/ml VEGF-A (R&D Systems, United Kingdom) with different concentrations of peptides (0.2, 1, 5, 25, and 125 μM), bevacizumab (Roche, Switzerland) at 6.5 μM (1 mg/ml) or control group (serum-free medium) were added and incubated for another 48 h (6 wells/concentration/group). The effects of proliferation were quantified by Cell Counting Kit-8 (CCK-8, Sigma, United States) assays. The absorbance was measured at 450 nm with an enzyme-linked immunoassay reader (AMR-100, ALLSHENG, Hangzhou, China). The experiment was repeated three times.

### *In Vivo* Antitumor Study of B-cL1 on Xenografted Mouse Model

The evaluation of antitumoral activity of B-cL1 was performed in Laboratory of Experimental Animal Science, Hangzhou Normal University (Hangzhou, China) maintained under standardized environmental conditions, with approved protocols by the Institutional Animal Care and Use Committee (IACUC) of Hangzhou Normal University. Human gastric cancer SGC-7901 cells (1 × 10^6^ cells/500 μL) from Cell Bank of the Chinese Academy of Sciences (Shanghai, China) were injected to the right flanks of 8 weeks old female BALB/c mice. When tumor size grown to 100–300 mm^3^, BALB/c mice were randomized to groups (*n* = 6). B-cL1 (5 mg/kg/day and 10 mg/kg/day) and PBS (control group) were intravenously administrated for 2 weeks, bevacizumab (5 mg/kg) was intravenously administrated only once during the 2 weeks as positive control. The tumor volume was measured every 2 days by a digital Vernier caliper, using the following formula: v = a^2^ × b × 0.52 (where a is the shortest diameter of tumor and b is the longest diameter of tumor). After 14 days of treatment, the mice were euthanized using CO_2_ followed by cervical dislocation to ensure death. Then, the tumors were separated, photographed and weighed on balance (OHAUS Adventure, United States).

### Statistical Analysis

Data are expressed as the arithmetic mean ± SEM of at least three different experiments using the GraphPad Prism software version 8.00 (San Diego, United States). The statistical significance of results was evaluated by one-way ANOVA, with probability values *p < 0.05, **p < 0.01, ***p < 0.001, ****p < 0.0001, being considered as significant, “ns” meaning non-significant.

## Conclusion

Our designed peptide B-cL1, mimicking Loop 1 of VEGF-B, showed significant anti-angiogenic and anti-tumor activities both *in vitro* and *in vivo*. B-cL1 can thus be considered as a peptide lead for anti-angiogenic drug research. The results in this study suggest targeting simultaneously D2 and D3 domains of VEGFR as a new concept to develop potent VEGFRs antagonists. The optimization of B-cL1 to achieve better dual binding peptides are under investigation.

Anti-VEGF therapeutic agents like antibodies have been generally used in combination with a cytotoxic agent. Recent studies demonstrate their beneficial use in combination with programmed cell death protein 1 (PD-1)/programmed cell death ligand 1 (PD-L1) interaction inhibitors in various solid tumor types (Gao and Yang, 2020). New peptides with different pharmacokinetic properties as compared to antibodies will be explored as potential drugs in combination with such inhibitors.

## Data Availability

The raw data supporting the conclusions of this article will be made available by the authors, without undue reservation.

## References

[B1] Aparicio-Gallego G. Blanco M. Figueroa A. García-Campelo R. Valladares-Ayerbes M. Grande-Pulido E. (2011). New Insights into Molecular Mechanisms of Sunitinib-Associated Side Effects. Mol. Cancer Ther. 10, 2215–2223. 10.1158/1535-7163.Mct-10-1124 22161785

[B2] Apte R. S. Chen D. S. Ferrara N. (2019). VEGF in Signaling and Disease: Beyond Discovery and Development. Cell 176, 1248–1264. 10.1016/j.cell.2019.01.021 30849371 PMC6410740

[B3] Assareh E. Mehrnejad F. Mansouri K. Esmaeili Rastaghi A. R. Naderi-Manesh H. Asghari S. M. (2019). A Cyclic Peptide Reproducing the α1 helix of VEGF-B Binds to VEGFR-1 and VEGFR-2 and Inhibits Angiogenesis and Tumor Growth. Biochem. J. 476, 645–663. 10.1042/BCJ20180823 30700502

[B4] Basile A. Del Gatto A. Diana D. Di Stasi R. Falco A. Festa M. (2011). Characterization of a Designed Vascular Endothelial Growth Factor Receptor Antagonist Helical Peptide with Antiangiogenic Activity *In Vivo* . J. Med. Chem. 54, 1391–1400. 10.1021/jm101435r 21280635

[B5] Bayó-Puxan N. Rodríguez-Mias R. Goldflam M. Kotev M. Ciudad S. Hipolito C. J. (2016). Combined Use of Oligopeptides, Fragment Libraries, and Natural Compounds: A Comprehensive Approach to Sample the Druggability of Vascular Endothelial Growth Factor. ChemMedChem 11, 928–939. 10.1002/cmdc.201500467 26553526 PMC5063151

[B6] Brozzo M. S. Bjelić S. Kisko K. Schleier T. Leppänen V. M. Alitalo K. (2012). Thermodynamic and Structural Description of Allosterically Regulated VEGFR-2 Dimerization. Blood 119, 1781–1788. 10.1182/blood-2011-11-390922 22207738

[B7] Cabral L. K. D. Tiribelli C. Sukowati C. H. C. (2020). Sorafenib Resistance in Hepatocellular Carcinoma: The Relevance of Genetic Heterogeneity. Cancers (Basel) 12, 1576. 10.3390/cancers12061576 PMC7352671 32549224

[B8] Carmeliet P. (2003). Angiogenesis in Health and Disease. Nat. Med. 9, 653–660. 10.1038/nm0603-653 12778163

[B9] Carmeliet P. Jain R. K. (2011). Molecular Mechanisms and Clinical Applications of Angiogenesis. Nature 473, 298–307. 10.1038/nature10144 21593862 PMC4049445

[B10] Choi J. S. Joo S. H. (2020). Recent Trends in Cyclic Peptides as Therapeutic Agents and Biochemical Tools. Biomol. Ther. (Seoul) 28, 18–24. 10.4062/biomolther.2019.082 31597413 PMC6939695

[B11] Christinger H. W. Fuh G. De Vos A. M. Wiesmann C. (2004). The crystal Structure of Placental Growth Factor in Complex with Domain 2 of Vascular Endothelial Growth Factor Receptor-1. J. Biol. Chem. 279, 10382–10388. 10.1074/jbc.M313237200 14684734

[B12] Chung A. S. Ferrara N. (2011). Developmental and Pathological Angiogenesis. Annu. Rev. Cel Dev Biol 27, 563–584. 10.1146/annurev-cellbio-092910-154002 21756109

[B13] De Rosa L. Diana D. Basile A. Russomanno A. Isernia C. Turco M. C. (2014). Design, Structural and Biological Characterization of a VEGF Inhibitor β-hairpin-constrained Peptide. Eur. J. Med. Chem. 73, 210–216. 10.1016/j.ejmech.2013.12.016 24412496

[B14] Diao L. Meibohm B. (2013). Pharmacokinetics and Pharmacokinetic-Pharmacodynamic Correlations of Therapeutic Peptides. Clin. Pharmacokinet. 52, 855–868. 10.1007/s40262-013-0079-0 23719681

[B15] Eelen G. Treps L. Li X. Carmeliet P. (2020). Basic and Therapeutic Aspects of Angiogenesis Updated. Circ. Res. 127, 310–329. 10.1161/CIRCRESAHA.120.316851 32833569

[B16] Faivre S. Demetri G. Sargent W. Raymond E. (2007). Molecular Basis for Sunitinib Efficacy and Future Clinical Development. Nat. Rev. Drug Discov. 6, 734–745. 10.1038/nrd2380 17690708

[B17] Farzaneh Behelgardi M. Zahri S. Mashayekhi F. Mansouri K. Asghari S. M. (2018). A Peptide Mimicking the Binding Sites of VEGF-A and VEGF-B Inhibits VEGFR-1/-2 Driven Angiogenesis, Tumor Growth and Metastasis. Sci. Rep. 8, 17924. 10.1038/s41598-018-36394-0 30560942 PMC6298961

[B18] Ferrara N. Gerber H. P. Lecouter J. (2003). The Biology of VEGF and its Receptors. Nat. Med. 9, 669–676. 10.1038/nm0603-669 12778165

[B19] Fosgerau K. Hoffmann T. (2015). Peptide Therapeutics: Current Status and Future Directions. Drug Discov. Today 20, 122–128. 10.1016/j.drudis.2014.10.003 25450771

[B20] Gao F. Yang C. (2020). Anti-VEGF/VEGFR2 Monoclonal Antibodies and Their Combinations with PD-1/pd-L1 Inhibitors in Clinic. Curr. Cancer Drug Targets 20, 3–18. 10.2174/1568009619666191114110359 31729943

[B21] Garcia J. Hurwitz H. I. Sandler A. B. Miles D. Coleman R. L. Deurloo R. (2020). Bevacizumab (Avastin®)in Cancer Treatment: A Review of 15 years of Clinical Experience and Future Outlook. Cancer Treat. Rev. 86, 102017. 10.1016/j.ctrv.2020.102017 32335505

[B22] García-Aranda M. I. González-López S. Santiveri C. M. Gagey-Eilstein N. Reille-Seroussi M. Martín-Martínez M. (2013). Helical Peptides from VEGF and Vammin Hotspots for Modulating the VEGF-VEGFR Interaction. Org. Biomol. Chem. 11, 1896–1905. 10.1039/c3ob27312a 23381088

[B23] Gautier B. Goncalves V. Diana D. Di Stasi R. Teillet F. Lenoir C. (2010). Biochemical and Structural Analysis of the Binding Determinants of a Vascular Endothelial Growth Factor Receptor Peptidic Antagonist. J. Med. Chem. 53, 4428–4440. 10.1021/jm1002167 20462213

[B24] Gayatri S. Cowles M. W. Vemulapalli V. Cheng D. Sun Z. W. Bedford M. T. (2016). Using Oriented Peptide Array Libraries to Evaluate Methylarginine-specific Antibodies and Arginine Methyltransferase Substrate Motifs. Sci. Rep. 6, 28718. 10.1038/srep28718 27338245 PMC4919620

[B25] Goncalves V. Gautier B. Coric P. Bouaziz S. Lenoir C. Garbay C. (2007a). Rational Design, Structure, and Biological Evaluation of Cyclic Peptides Mimicking the Vascular Endothelial Growth Factor. J. Med. Chem. 50, 5135–5146. 10.1021/jm0706970 17900101

[B26] Goncalves V. Gautier B. Garbay C. Vidal M. Inguimbert N. (2007b). Development of a Chemiluminescent Screening Assay for Detection of Vascular Endothelial Growth Factor Receptor 1 Ligands. Anal. Biochem. 366, 108–110. 10.1016/j.ab.2007.03.027 17482136

[B27] Hanahan D. Folkman J. (1996). Patterns and Emerging Mechanisms of the Angiogenic Switch during Tumorigenesis. Cell 86, 353–364. 10.1016/s0092-8674(00)80108-7 8756718

[B28] Henninot A. Collins J. C. Nuss J. M. (2018). The Current State of Peptide Drug Discovery: Back to the Future?. J. Med. Chem. 61, 1382–1414. 10.1021/acs.jmedchem.7b00318 28737935

[B29] Iyer S. Darley P. I. Acharya K. R. (2010). Structural Insights into the Binding of Vascular Endothelial Growth Factor-B by VEGFR-1(D2): Recognition and Specificity. J. Biol. Chem. 285, 23779–23789. 10.1074/jbc.M110.130658 20501651 PMC2911289

[B30] Klotz L. Norman S. Vieira J. M. Masters M. Rohling M. Dubé K. N. (2015). Cardiac Lymphatics Are Heterogeneous in Origin and Respond to Injury. Nature 522, 62–67. 10.1038/nature14483 25992544 PMC4458138

[B31] Kong D. H. Kim M. R. Jang J. H. Na H. J. Lee S. (2017). A Review of Anti-angiogenic Targets for Monoclonal Antibody Cancer Therapy. Int. J. Mol. Sci. 18, 1786. 10.3390/ijms18081786 PMC5578174 28817103

[B32] Lazzeri S. Ripandelli G. Sartini M. S. Parravano M. Varano M. Nardi M. (2015). Aflibercept Administration in Neovascular Age-Related Macular Degeneration Refractory to Previous Anti-vascular Endothelial Growth Factor Drugs: a Critical Review and New Possible Approaches to Move Forward. Angiogenesis 18, 397–432. 10.1007/s10456-015-9483-4 26346237

[B33] London N. Raveh B. Cohen E. Fathi G. Schueler-Furman O. (2011). Rosetta FlexPepDock Web Server-Hhigh Resolution Modeling of Peptide-Protein Interactions. Nucleic Acids Res. 39, W249–W253. 10.1093/nar/gkr431 21622962 PMC3125795

[B34] Lv Y. Zhou D. Hao X. Q. Zhu M. Y. Zhang C. D. Zhou D. M. (2019). A Recombinant Measles Virus Vaccine Strain rMV-Hu191 Has Oncolytic Effect against Human Gastric Cancer by Inducing Apoptotic Cell Death Requiring Integrity of Lipid Raft Microdomains. Cancer Lett. 460, 108–118. 10.1016/j.canlet.2019.06.010 31226409

[B35] Markovic-Mueller S. Stuttfeld E. Asthana M. Weinert T. Bliven S. Goldie K. N. (2017). Structure of the Full-Length VEGFR-1 Extracellular Domain in Complex with VEGF-A. Structure 25, 341–352. 10.1016/j.str.2016.12.012 28111021

[B36] Morais C. (2014). Sunitinib Resistance in Renal Cell Carcinoma. J. Kidney Cancer VHL 1, 1–11. 10.15586/jkcvhl.2014.7 28326244 PMC5345511

[B37] Moserle L. Jiménez-Valerio G. Casanovas O. (2014). Antiangiogenic Therapies: Going beyond Their Limits. Cancer Discov. 4, 31–41. 10.1158/2159-8290.Cd-13-0199 24356098

[B38] Ng E. W. Shima D. T. Calias P. Cunningham E. T. Jr. Guyer D. R. Adamis A. P. (2006). Pegaptanib, a Targeted Anti-VEGF Aptamer for Ocular Vascular Disease. Nat. Rev. Drug Discov. 5, 123–132. 10.1038/nrd1955 16518379

[B39] Paci A. Desnoyer A. Delahousse J. Blondel L. Maritaz C. Chaput N. (2020). Pharmacokinetic/pharmacodynamic Relationship of Therapeutic Monoclonal Antibodies Used in Oncology: Part 1, Monoclonal Antibodies, Antibody-Drug Conjugates and Bispecific T-Cell Engagers. Eur. J. Cancer 128, 107–118. 10.1016/j.ejca.2020.01.005 32037061

[B40] Park D. J. Thomas N. J. Yoon C. Yoon S. S. (2015). Vascular Endothelial Growth Factor a Inhibition in Gastric Cancer. Gastric Cancer 18, 33–42. 10.1007/s10120-014-0397-4 24993497

[B41] Potente M. Gerhardt H. Carmeliet P. (2011). Basic and Therapeutic Aspects of Angiogenesis. Cell 146, 873–887. 10.1016/j.cell.2011.08.039 21925313

[B42] Raveh B. London N. Schueler-Furman O. (2010). Sub-angstrom Modeling of Complexes between Flexible Peptides and Globular Proteins. Proteins 78, 2029–2040. 10.1002/prot.22716 20455260

[B43] Reille-Seroussi M. Gaucher J. F. Desole C. Gagey-Eilstein N. Brachet F. Broutin I. (2015). Vascular Endothelial Growth Factor Peptide Ligands Explored by Competition Assay and Isothermal Titration Calorimetry. Biochemistry 54, 5147–5156. 10.1021/acs.biochem.5b00722 26222917

[B44] Rezzola S. Loda A. Corsini M. Semeraro F. Annese T. Presta M. (2020). Angiogenesis-Inflammation Cross Talk in Diabetic Retinopathy: Novel Insights from the Chick Embryo Chorioallantoic Membrane/Human Vitreous Platform. Front. Immunol. 11, 581288. 10.3389/fimmu.2020.581288 33117388 PMC7552803

[B45] Sadremomtaz A. Ali A. M. Jouyandeh F. Balalaie S. Navari R. Broussy S. (2020). Molecular Docking, Synthesis and Biological Evaluation of Vascular Endothelial Growth Factor (VEGF) B Based Peptide as Antiangiogenic Agent Targeting the Second Domain of the Vascular Endothelial Growth Factor Receptor 1 (VEGFR1D2) for Anticancer Application. Signal. Transduct Target. Ther. 5, 76. 10.1038/s41392-020-0177-z 32499505 PMC7272640

[B46] Sadremomtaz A. Mansouri K. Alemzadeh G. Safa M. Rastaghi A. E. Asghari S. M. (2018). Dual Blockade of VEGFR1 and VEGFR2 by a Novel Peptide Abrogates VEGF-Driven Angiogenesis, Tumor Growth, and Metastasis through PI3K/AKT and MAPK/ERK1/2 Pathway. Biochim. Biophys. Acta Gen. Subj 1862, 2688–2700. 10.1016/j.bbagen.2018.08.013 30251659

[B47] Sawaguchi S. Varshney S. Ogawa M. Sakaidani Y. Yagi H. Takeshita K. (2017). O-GlcNAc on NOTCH1 EGF Repeats Regulates Ligand-Induced Notch Signaling and Vascular Development in Mammals. Elife 6, e24419. 10.7554/eLife.24419 28395734 PMC5388531

[B48] Shibuya M. Claesson-Welsh L. (2006). Signal Transduction by VEGF Receptors in Regulation of Angiogenesis and Lymphangiogenesis. Exp. Cel Res 312, 549–560. 10.1016/j.yexcr.2005.11.012 16336962

[B49] Shibuya M. (2011). Vascular Endothelial Growth Factor (VEGF) and its Receptor (VEGFR) Signaling in Angiogenesis: A Crucial Target for Anti- and Pro-angiogenic Therapies. Genes Cancer 2, 1097–1105. 10.1177/1947601911423031 22866201 PMC3411125

[B50] Shibuya M. (2014). VEGF-VEGFR Signals in Health and Disease. Biomol. Ther. (Seoul) 22, 1–9. 10.4062/biomolther.2013.113 24596615 PMC3936422

[B51] Sun H.-C. Zhang W. Zhu X.-D. Lu L. Zhang Q.-B. Tang Z.-Y. (2013). Abstract 5086: The Side Effects of Sorafenib in Treating Hepatocellular Carcinoma. Cancer Res. 73, 5086. 10.1158/1538-7445.Am2013-5086

[B52] Van Geel R. M. Beijnen J. H. Schellens J. H. (2012). Concise Drug Review: Pazopanib and Axitinib. Oncologist 17, 1081–1089. 10.1634/theoncologist.2012-0055 22733795 PMC3425526

[B53] Vasudev N. S. Reynolds A. R. (2014). Anti-angiogenic Therapy for Cancer: Current Progress, Unresolved Questions and Future Directions. Angiogenesis 17, 471–494. 10.1007/s10456-014-9420-y 24482243 PMC4061466

[B54] Wang L. Coric P. Broussy S. Di Stasi R. Zhou L. D'andrea L. D. (2019). Structural Studies of the Binding of an Antagonistic Cyclic Peptide to the VEGFR1 Domain 2. Eur. J. Med. Chem. 169, 65–75. 10.1016/j.ejmech.2019.02.069 30856407

[B55] Wang L. Gagey-Eilstein N. Broussy S. Reille-Seroussi M. Huguenot F. Vidal M. (2014). Design and Synthesis of C-Terminal Modified Cyclic Peptides as VEGFR1 Antagonists. Molecules 19, 15391–15407. 10.3390/molecules191015391 25264829 PMC6270838

[B56] Wang L. Zhou L. Reille-Seroussi M. Gagey-Eilstein N. Broussy S. Zhang T. (2017). Identification of Peptidic Antagonists of Vascular Endothelial Growth Factor Receptor 1 by Scanning the Binding Epitopes of its Ligands. J. Med. Chem. 60, 6598–6606. 10.1021/acs.jmedchem.7b00283 28686443

[B57] Wiesmann C. Fuh G. Christinger H. W. Eigenbrot C. Wells J. A. De Vos A. M. (1997). Crystal Structure at 1.7 A Resolution of VEGF in Complex with Domain 2 of the Flt-1 Receptor. Cell 91, 695–704. 10.1016/s0092-8674(00)80456-0 9393862

[B58] Wilhelm S. Carter C. Lynch M. Lowinger T. Dumas J. Smith R. A. (2006). Discovery and Development of Sorafenib: a Multikinase Inhibitor for Treating Cancer. Nat. Rev. Drug Discov. 5, 835–844. 10.1038/nrd2130 17016424

[B59] Zudaire E. Gambardella L. Kurcz C. Vermeren S. (2011). A Computational Tool for Quantitative Analysis of Vascular Networks. PLoS One 6, e27385. 10.1371/journal.pone.0027385 22110636 PMC3217985

