# Supplementary material for: A Cyclic Peptide Epitope of an Under-Explored VEGF-B Loop 1 Demonstrated In Vivo Anti-Angiogenic and Anti-Tumor Activities
Source: Front Pharmacol. 2021 Sep 29;12:734544. doi: 10.3389/fphar.2021.734544 (PMC8511632; doi:10.3389/fphar.2021.734544)
Supplement: Supplementary file 1 [file DataSheet1.docx]

Supplementary Material

# Supplementary Figures and Tables

**Docking of A-cL1, B-cL1 and P-cL1 in binding with VEGFR1.**

| 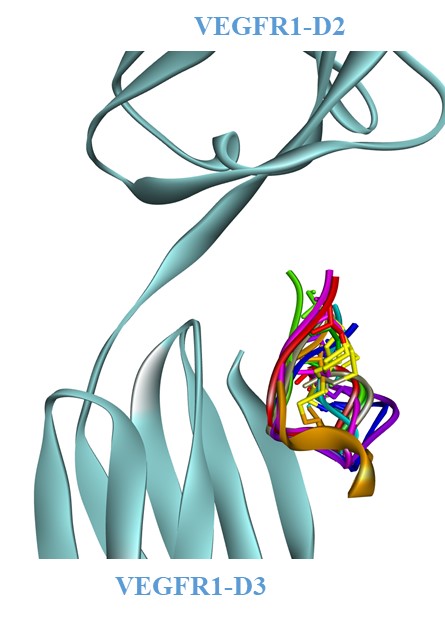  **Figure S1-A**. Superimpose of 10 structures with highest score of A-cL1 in binding with VEGFR1. | \| Structure ID: A-cL1 \| Total-score \| \| --- \| --- \| \| Top1 \| -372.123 \| \| Top2 \| -369.984 \| \| Top3 \| -368.788 \| \| Top4 \| -367.850 \| \| Top5 \| -366.788 \| \| Top6 \| -366.787 \| \| Top7 \| -366.293 \| \| Top8 \| -366.159 \| \| Top9 \| -366.103 \| \| Top10 \| -365.106 \|   **Table S1**. Highest scores of 10 optimized structure models of peptide A-cL1 with VEGFR1 calculated by Rosetta FlexPepDock program. |
| --- | --- | --- | --- | --- | --- | --- | --- | --- | --- | --- | --- | --- | --- | --- | --- | --- | --- | --- | --- | --- | --- | --- | --- |
| 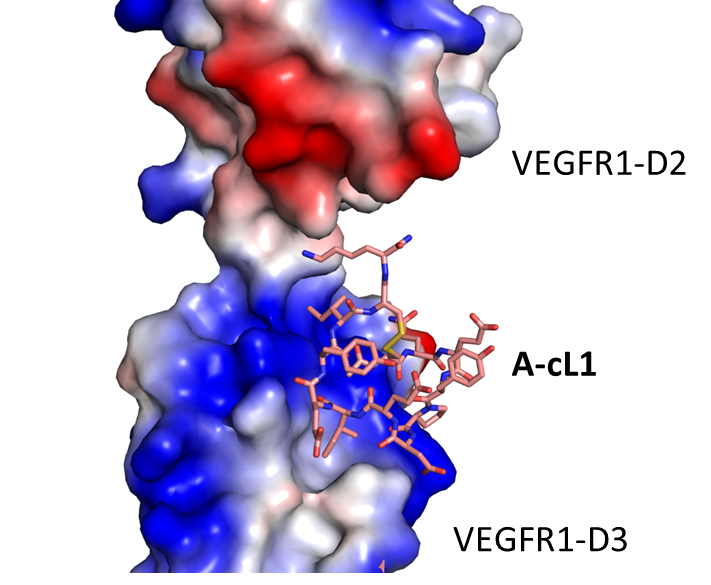  **Figure S1-B.** Structure of A-cL1 with highest score in binding with VEGFR1. | |

| 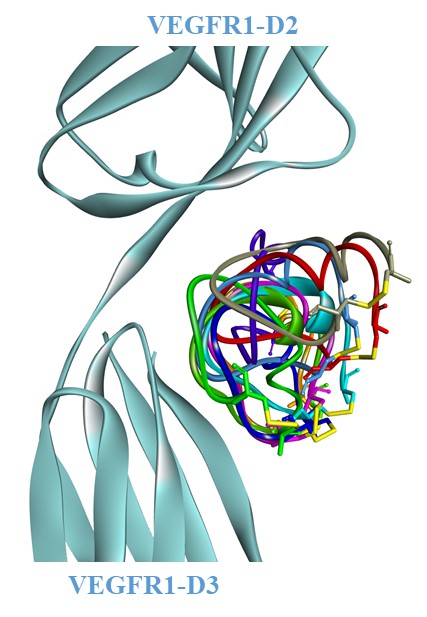  **Figure S2-A**. Superimpose of ten structures with highest score of B-cL1 in binding with VEGFR1. | \| Structure ID:  B-cL1 \| Total-score \| \| --- \| --- \| \| Top1 \| -347.965 \| \| Top2 \| -346.896 \| \| Top3 \| -346.194 \| \| Top4 \| -343.909 \| \| Top5 \| -342.963 \| \| Top6 \| -342.130 \| \| Top7 \| -342.056 \| \| Top8 \| -341.991 \| \| Top9 \| -341.933 \| \| Top10 \| -341.812 \|   **Table S2**. Highest scores of ten optimized structure models of peptide B-cL1 with VEGFR1 calculated by Rosetta FlexPepDock program. |
| --- | --- | --- | --- | --- | --- | --- | --- | --- | --- | --- | --- | --- | --- | --- | --- | --- | --- | --- | --- | --- | --- | --- | --- |
| 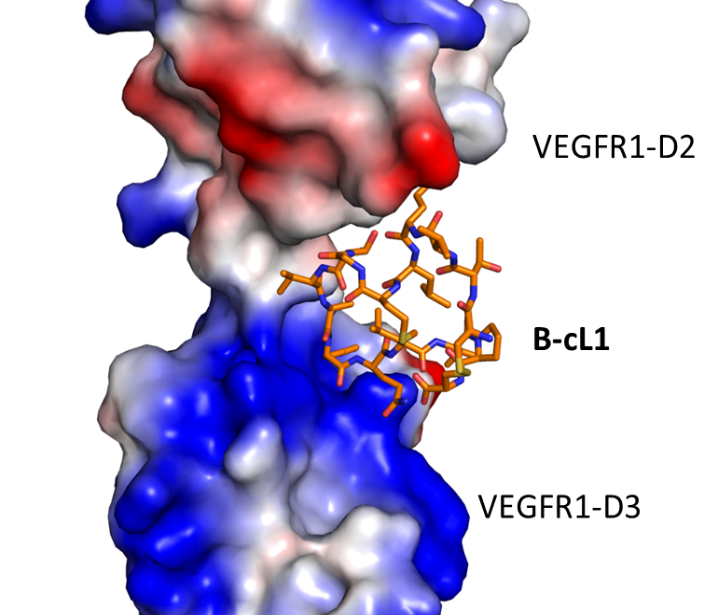  **Figure S2-B.** Structure of B-cL1 with highest score in binding with VEGFR1. | |

| 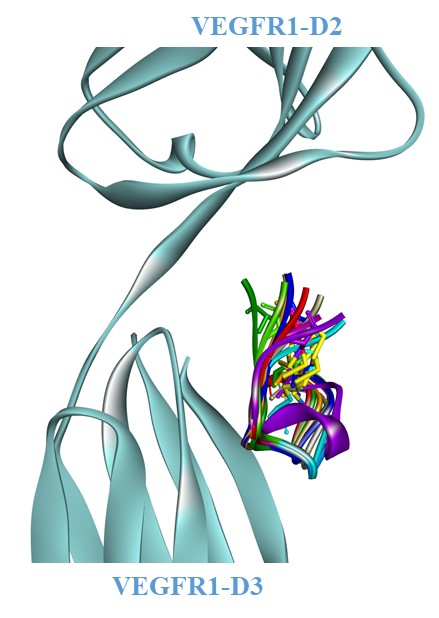  **Figure S3-A**. Superimpose of ten structures with highest score of P-cL1 in binding with VEGFR1. | \| Structure ID: P-cL1 \| Total-score \| \| --- \| --- \| \| Top1 \| -376.486 \| \| Top2 \| -375.571 \| \| Top3 \| -374.120 \| \| Top4 \| -373.805 \| \| Top5 \| -373.020 \| \| Top6 \| -372.984 \| \| Top7 \| -371.557 \| \| Top8 \| -371.372 \| \| Top9 \| -370.435 \| \| Top10 \| -370.195 \|   **Table S3**. Highest scores of ten optimized structure models of peptide P-cL1 with VEGFR1 calculated by Rosetta FlexPepDock program. |
| --- | --- | --- | --- | --- | --- | --- | --- | --- | --- | --- | --- | --- | --- | --- | --- | --- | --- | --- | --- | --- | --- | --- | --- |
| **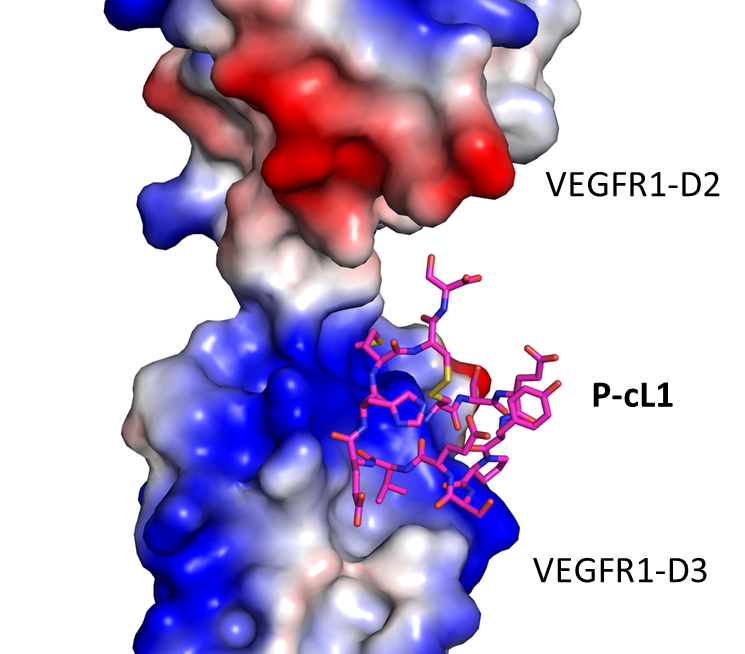**  **Figure S3-B**. Structure of P-cL1 with highest score in binding with VEGFR1. | |

**Scheme S1. Formulas of A-cL1, B-cL1 and P-cL1**

| **A-cL1**  Sequence  [_1_CQEYPDEIEYIC]K_13_ |  |
| --- | --- |
| **B-cL1**  Sequence  Ac-[_1_CTVELMGTVAKQLVPC_16_] | **** |
| **P-cL1**  Sequence  [_1_CSEYPSEVEHMC]S_13_ |  |
